# A comprehensive evaluation of food fortification with folic acid for the primary prevention of neural tube defects

**DOI:** 10.1186/1471-2393-4-20

**Published:** 2004-09-27

**Authors:** Shiliang Liu, Roy West, Edward Randell, Linda Longerich, Kathleen Steel O'Connor, Helen Scott, Marian Crowley, Angeline Lam, Victor Prabhakaran, Catherine McCourt

**Affiliations:** 1 Health Surveillance and Epidemiology Division, Centre for Healthy Human Development, PPHB, Health Canada, Ottawa, Ontario, Canada; 2 Division of Community Health, Faculty of Medicine, Memorial University, St. John's, Newfoundland and Labrador, Canada; 3 Health Sciences Centre and Division of Laboratory Medicine; Faculty of Medicine, Memorial University, St. John's, Newfoundland and Labrador, Canada; 4 Public Health Research, Education and Development Program, Kingston, Frontenac and Lennox & Addington Health Unit, Kingston, Ontario, Canada; 5 Department of Public Health Sciences, University of Toronto, Toronto, Ontario, Canada; 6 Provincial Medical Genetics Program, Health Care Corporation of St. John's, St. John's, Newfoundland and Labrador, Canada; 7 Department of Earth Sciences, University of Waterloo, Waterloo, Ontario, Canada; 8 London Health Sciences Centre & Department of Clinical Biochemistry, University of Western Ontario, London, Ontario, Canada

## Abstract

**Background:**

Periconceptional use of vitamin supplements containing folic acid reduces the risk of a neural tube defect (NTD). In November 1998, food fortification with folic acid was mandated in Canada, as a public health strategy to increase the folic acid intake of all women of childbearing age. We undertook a comprehensive population based study in Newfoundland to assess the benefits and possible adverse effects of this intervention.

**Methods:**

This study was carried out in women aged 19–44 years and in seniors from November 1997 to March 1998, and from November 2000 to March 2001. The evaluation was comprised of four components: I) Determination of rates of NTDs; II) Dietary assessment; III) Blood analysis; IV) Assessment of knowledge and use of folic acid supplements.

**Results:**

The annual rates of NTDs in Newfoundland varied greatly between 1976 and 1997, with a mean rate of 3.40 per 1,000 births. There was no significant change in the average rates between 1991–93 and 1994–97 (relative risk [RR] 1.01, 95% confidence interval [CI] 0.76–1.34). The rates of NTDs fell by 78% (95% CI 65%–86%) after the implementation of folic acid fortification, from an average of 4.36 per 1,000 births during 1991–1997 to 0.96 per 1,000 births during 1998–2001 (RR 0.22, 95% CI 0.14–0.35). The average dietary intake of folic acid due to fortification was 70 μg/day in women aged 19–44 years and 74 μg/day in seniors. There were significant increases in serum and RBC folate levels for women and seniors after mandatory fortification. Among seniors, there were no significant changes in indices typical of vitamin B_12 _deficiencies, and no evidence of improved folate status masking haematological manifestations of vitamin B_12 _deficiency. The proportion of women aged 19–44 years taking a vitamin supplement containing folic acid increased from 17% to 28%.

**Conclusions:**

Based on these findings, mandatory food fortification in Canada should continue at the current levels. Public education regarding folic acid supplement use by women of childbearing age should also continue.

## Background

Neural tube defects (NTDs) are birth defects resulting from the failure of neural tube closure during early development of the human embryo. The 1997 Canadian national NTD birth prevalence was 0.75 per 1,000 births (live births and stillbirths), down from 1.16 per 1,000 in 1989 [1]. The rates tend to be higher in the eastern provinces than in the west [2-4]. Historically, Newfoundland has had one of the highest rates in North America with a reported average yearly rate for 1976–1997 of 3.4 per 1,000 births (including live births, stillbirths and fetuses from pregnancies terminated after a prenatal diagnosis of an NTD) [4].

Evidence from a number of studies has demonstrated that periconceptional use of vitamin supplements containing folic acid reduces the risk of NTDs [5-8]. Although the mechanism of action of this nutrient in influencing the risk of NTDs is poorly understood, the evidence of the benefit of folic acid has led many health organizations since late 1992 to recommend periconceptional folic acid supplementation, at a level of 400 μg /day for low risk women [9-11].

Because of concern that public education campaigns alone would not be effective in achieving optimal periconceptional folic acid intake for the majority of women, food fortification with folic acid was proposed as a strategy to ensure that all women of childbearing age increase their dietary intake of this vitamin. In November 1998, Health Canada mandated fortification of white flour and enriched pasta and cornmeal with folic acid [12]. Since diets vary, it was known that it would be virtually impossible to fortify food with folic acid at a level that ensures that the target population receives an additional 400 μg /day, while protecting the non-targeted population from an undesirably high amount. As a result, conservative levels of fortification were introduced. White flour is fortified with folic acid at a level of 0.15 mg per 100 g of flour. This intervention was expected to increase the average daily folic acid intake of women of childbearing age by about 100 μg [13].

The question of whether folic acid fortification of grain products poses any serious health risk has been controversial. The main concern has been the potential masking of vitamin B_12 _deficiency, a condition that affects 10–15% of the population over age 60 years [14,15]. Increased folic acid intake may correct the haematologic signs of vitamin B_12 _deficiency, thus delaying diagnosis and treatment of the condition while its attendant neurologic manifestations progress. Seniors may be at particular risk since the incidence of vitamin B_12 _deficiency increases with age.

We therefore undertook a comprehensive population based study to evaluate the effectiveness of the public health strategy of food fortification with folic acid and to determine possible adverse effects resulting from fortification.

## Methods

### Study design

This evaluative study was designed as a population based study and included four components as follows: I) Determination of rates of NTDs; II) Dietary assessment; III) Blood analysis; IV) Assessment of knowledge and use of folic acid supplements. The latter three components of the study were carried out in two phases; the first phase took place prior to the introduction of mandatory fortification, from November 1997 to March 1998 and the second phase occurred from November 2000 to March 2001, after two years of implementation of mandatory fortification.

This study was undertaken in Newfoundland because of the historically high rates of NTDs in the province, and because of strong interest in the health community in this initiative. Newfoundland and Labrador, with a population of approximately 500,000, has about 5,000 births annually. An urban (St. John's) and rural (Clarenville, Port Blandford, Random Island area) location in the province were chosen as the sites for this study. Data collected from these sites were compared between Phase I (November 1997 to March 1998) and Phase II (November 2000 to March 2001). Table 1 shows schematically the framework including objectives and sampling of subjects for this study. As part of this project, dietary assessment, blood analysis and assessment of knowledge and use of supplements were also carried out in a 2-phase population based study of women of reproductive age in Kingston, Ontario and environs. The results of this study will be reported elsewhere.

**Table 1:** Framework for a two phase, multi site study to examine the effects of food fortification with folic acid

Content	Study objective	Sample **	Location
I. Rates of NTDs	Determine rate of NTD-affected pregnancies, pre and post fortification	Newfoundland population	Newfoundland
II. Dietary assessment*	Determine dietary intake of folate, pre and post Fortification	A) Non-pregnant women of childbearing age (19–44 years), not taking supplements containing folic acid;	St. John's, Rural Newfoundland
III. Blood analysis*	Determine blood folate and vitamin B_12 _status, pre and post fortification	B) Seniors (65 years or older) not taking supplements containing folic acid or B_12 _supplement and not diagnosed with anaemia.	St. John's, Rural Newfoundland
IV. Knowledge and intake of folic acid supplements	Determine knowledge and consumption of folic acid supplements, pre and post fortification	Non-pregnant women of childbearing age (19–44 years)	St. John's, Rural Newfoundland

* The same sample of women and seniors are analyzed in Components II and III.

** Sampling was done separately for Phases I and II.

### Data collection

In order to examine temporal changes in the rates of NTDs in Newfoundland, data were compiled from the Newfoundland and Labrador Medical Genetics Program from 1976 to 2001. This Program ascertains cases of NTDs annually and maintains an NTD database. The database has recorded cases of NTD since 1976. Cases are identified in the following ways: provincial live birth and stillbirth notification forms, maternal-fetal medicine referrals (only one tertiary care unit in the province), and letters sent to all medical records departments of all provincial hospitals requesting data on cases assigned ICD-9/10 codes associated with NTDs or terminations for NTDs. These multiple sources are utilized to ensure complete ascertainment. NTD cases include anencephaly, spina bifida and encephalocele diagnosed in live births, stillbirths (a gestational age of 20 weeks and above or birthweight of 500 g and above) and fetuses from pregnancies terminated (at any gestational age) after a prenatal diagnosis of an NTD.

For the knowledge assessment component of the study, women between the ages of 19 and 44 years were recruited through a random telephone survey. In the initial telephone survey, women were asked about their use of vitamin supplements and knowledge of the importance of folic acid for reducing the risk of NTDs or for fetal development. Women who completed the initial telephone survey were subsequently screened for their eligibility for dietary and blood assessments. Women who were not taking supplements containing folic acid and not pregnant were eligible to participate. This sampling procedure for Phase I and Phase II resulted in a response rate of 59.7% and 65.4%, respectively, with no difference between urban and rural response rates. A total of 233 women were recruited into Phase I and 204 women were recruited in Phase II, who completed components II, III and IV of the study.

Seniors were recruited in the same manner as the samples of women, but were drawn only from St. John's, Newfoundland. Seniors aged 65 years or over, not diagnosed with vitamin B_12 _deficiency or anaemia and not taking vitamin B_12 _or supplements containing folic acid, were eligible for dietary and blood sample assessments. A total of 202 seniors were recruited in Phase I and 186 were recruited in Phase II (response rate 45.1% and 44.9%, respectively).

In order to determine intakes of naturally occurring folate (the form of the vitamin found naturally in foods) pre and post fortification, and dietary intakes of folic acid (the synthetic form of the vitamin) post fortification, a Willett food frequency dietary questionnaire [16] was administered to subjects during an in-person interview. There were some modifications to the questionnaire to include common Newfoundland foods and to ensure that all foods high in folate were included. The dietary questionnaire was used to estimate an average frequency of consumption of 124 food items over the previous period of one year.

The women and senior participants were also asked to provide a sample of blood in order to determine blood folate and vitamin B_12 _status in Phase I and Phase II. Laboratory tests for complete blood count (CBC), red blood cell (RBC) folate, serum folate, creatinine, vitamin B_12_, plasma homocysteine (HCY) and methylmalonic acid (MMA) were conducted at the laboratories of the Health Care Corporation of St. John's.

### Data analysis

Rate of NTDs was defined as the number of above described NTD cases, divided by the total number of live births, stillbirths, and pregnancy terminations for an NTD (termed as "births" hereafter). First we examined the temporal trend in annual rates of NTDs from 1976 to 2001 using 3-year moving average rates, then we focused on comparison of the NTD data for the most recent 11 years, identified as pre-supplementation (1991–1993), pre-fortification (1994–1997) and post-fortification (1998–2001). We regard the year 1997 as a transition period, or partial fortification period, since fortification of white flour and enriched pasta and cornmeal was *permitted *in Canada as of December 1996 [17]. Thus we also analysed the NTD data using 1994–1996 as a pre-fortification period.

Mean daily intakes of naturally occurring folate were calculated for women aged 19–44 years and for seniors in Phase I and Phase II. Also, for Phase II, average daily intakes of folic acid from fortified foods were calculated.

Data from the blood analyses were tested for normality with the Komogorov-Smirnov test, and differences between groups were tested using the non-parametric Mann-Whitney U test. The distributions of plasma MMA, plasma HCY, serum folate, RBC folate and serum vitamin B_12_were skewed. Values were therefore log transformed to give an approximate normal distribution for estimation of geometric mean and confidence intervals. Unless otherwise stated, all laboratory values presented in this paper are geometric means and 95% confidence intervals (CI). Differences in the frequency of high or low results based on reference values were tested by Pearson chi-square statistics.

All data for this study were entered into SPSS (the Statistical Package for Social Sciences) Rel. 10.0 after the end of each phase. Data from the dietary interviews were analyzed using Epi-Info (Version 6.04d), while the laboratory data and the data about knowledge and use of supplements were analyzed using SPSS.

## Results

### 1. Rate of NTDs

**Figure 1:**
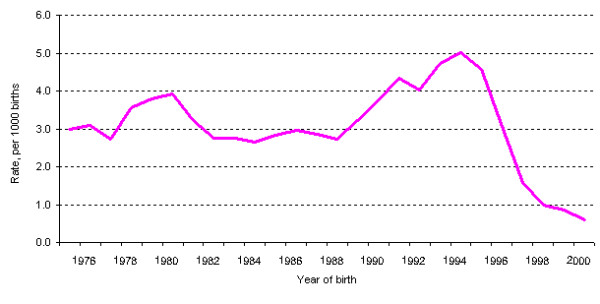
Rates of NTDs in Newfoundland and Labrador, 1976 to 2001 (3-year moving average rates) *The rate for 1976 is a 2-yr average based on data for 1976 and 1977 and the rate for 2001 is a 2-yr average based on data for 2000 and 2001.

There were 617 ascertained cases of NTD among live births, stillbirths and pregnancies terminated for an NTD in Newfoundland over the 26 year period. The annual rates of NTDs in the province varied greatly over time, with the lowest rate of 2.18 per 1,000 births in 1989, and the highest rate of 5.92 per 1,000 births in 1995. The average rate of NTDs between 1976 and 1997 was 3.40 per 1,000 births. A dramatic drop is seen in 1997, in which the rate of NTDs was 2.20 per 1,000 births, down from 5.49 per 1,000 births in the previous year. The decreasing trend continued after 1998 (Figure 1 shows 3-year moving average rates).

The NTD data for the years 1991–2001 are presented in three periods in Table 2. The mean annual rates were 4.35 per 1,000 births during 1991–1993 and 5.02 per 1,000 births during 1994–1996 (1994–96 vs 1991–93, relative risk [RR] 1.15, 95% CI 0.86–1.54, p = 0.95), and 4.37 per 1,000 births during 1994–1997 (1994–97 vs 1991–93, RR 1.01, 95% CI 0.76–1.34, p = 0.54).

**Table 2:** Annual rates of neural tube defects (NTDs) in Newfoundland and Labrador before folic acid supplementation (1991–1993), prior to folic acid fortification (1994–1997) and after fortification (1998–2001)

Period	No. of cases of NTDs			Total no. of births*	Rate per 1,000 births
	In live births and stillbirths	In terminated pregnancies	Total		
Pre-supplementation					
1991–1993	50	40	90	20,711	4.35
Pre-fortification					
1994–1997	53	50	103	23,592	4.37
Post-fortification					
1998–2001	8	11	19	19,816	0.96

* The total number of births includes live births, stillbirths and terminations for an NTD.

The total annual rate of NTDs fell by 78% after the implementation of folic acid fortification, from an average of 4.36 per 1,000 births during 1991–1997 to 0.96 per 1,000 births during 1998–2001 (RR 0.22, 95% CI 0.14–0.35, p < 0.0001). It is worthwhile to note that there has been no significant increase in the proportion of NTDs from terminated pregnancies since 1994.

### II. Dietary Assessment

There was no statistically significant change in the average daily intake of naturally occurring folate among either women aged 19–44 years or seniors between Phase I and Phase II (p = 0.19 and p = 0.18, respectively). Seniors generally had dietary folate intake slightly higher than women of childbearing age. In Phase I, the average daily intake of naturally occurring folate was 306 μg/day for seniors and 262 μg/day for women aged 19–44 years, while in Phase II, the average daily intake of naturally occurring folate was 290 μg/day for seniors and 248 μg/day for women aged 19–44 years.

The implementation of mandatory fortification resulted in an average additional dietary intake of 70 μg/day of folic acid in women aged 19–44, and 74 μg/day of folic acid among seniors. It is noteworthy that for the women the average daily folic acid intake due to food fortification was less than the approximately 100 μg that was previously predicted for women of childbearing age. The maximum dietary intake of folic acid due to fortification for an individual woman was 235 μg/day, and for an individual senior was 219 μg/day.

### III. Blood Analysis

Serum folate and RBC folate increased significantly from Phase I to Phase II in both women aged 19–44 years and seniors (p < 0.001). For both age groups, there was a corresponding decrease in mean plasma HCY levels (Tables 3 and 4). The prevalence of low serum folate (≤6.8 nmol/L) was eliminated from the sample of seniors and the proportion of elderly participants with low stores as indicated by RBC folate levels (< 373 nmol/L) was reduced from 2.5% to 1.6%. The proportion of women aged 19–44 years with high HCY(>13.2 μmol/L) also decreased from 15.9% to 7.6% (p = 0.002) (data not shown).

**Table 3:** Laboratory data (geometric mean and 95% confidence interval) for young women participants (age 19–44 years) in Phase I and Phase II

Characteristic	Phase I	Phase II	p value ^†^
Total participants (n)	233	204	
Serum folate (nmol/L)	13.5 (12.9 – 14.1)	18.1 (17.3 – 18.9)	<0.001
RBC folate (mol/L)	625 (601 – 649)	818 (784 – 854)	<0.001
Plasma HCY (μmol/L)	10.2 (9.8 – 10.7)	9.2 (8.8 – 9.6)	0.001
Serum vitamin B_12 _(pmol/L)	177 (169 – 186)	200 (190 – 211)	0.02
Plasma MMA (μmol/L)	0.18 (0.17 – 0.19)	0.21 (0.19 – 0.22)	0.008

† P value for the difference between Phase I and Phase II is based on a non-parametric Mann-Whitney U test.

RBC denotes red blood cell, HCY denotes homocysteine, and MMA refers to methylmalonic acid.

**Table 4:** Laboratory data (geometric mean and 95% confidence interval) for senior participants (age 65 years or over) between Phase I and Phase II

Characteristic	Phase I	Phase II	P value ^‡^
Total participants (n)	202	186	
Serum folate (nmol/L)	14.8 (14.0 – 15.6)	23.0 (22.0 – 24.1)	<0.001
RBC folate (mol/L)	745 (713 – 779)	916 (873 – 961)^†^	<0.001
Plasma HCY (μmol/L)	13.6 (13.0 – 14.2)^†^	12.3 (11.7 – 12.9)^†^	0.001
Serum vitamin B_12 _(pmol/L)	183 (173 – 194)	216 (202 – 231)	<0.001
Plasma MMA (μmol/L)	0.24 (0.22 – 0.27)^†^	0.26 (0.24 – 0.28)	0.229

† The Komogorov-Smirnov test showed that the log transformed data were non-normal (p < 0.05).

‡ P value for the difference between Phase I and Phase II is based on a non-parametric Mann-Whitney U test.

RBC denotes red blood cell, HCY denotes homocysteine and MMA refers to methylmalonic acid.

There was a significant increase in mean vitamin B_12 _levels in women aged 19–44 and seniors (p = 0.020 and p < 0.001, respectively, Tables 3 and 4). The proportion of seniors with low vitamin B_12 _(<133 pmol/L) was 18.8% prior to fortification and following fortification this proportion declined to 11.8% (p = 0.032) (data not shown). A statistically significant increase in mean plasma MMA levels was observed in women subjects (p = 0.008) but not in seniors (p = 0.229) (Tables 3 and 4). There was also an increase in the proportion of women aged 19–44 years with MMA values above the upper reference value of 0.37 μmol/L from 3.6% in Phase I to 14.9% in Phase II (p < 0.001). There was no significant change in the proportion of abnormal MMA values in seniors. Moreover, among seniors, blood analysis showed no significant difference in mean haemoglobin concentrations, mean corpuscular volume (MCV), or proportion with abnormally high MCV (>99 fL) or low haemoglobin (<120 g/L) concentrations.

### IV. Knowledge and use of folic acid supplements

There was a significant increase from Phase I to Phase II in the proportion of women aged 19–44 years who knew the importance of folic acid (from 33% to 46%, p < 0.001). The proportion of women taking a vitamin supplement containing folic acid increased substantially between the two time periods (from 17% to 28%, p < 0.003). Information about folic acid dosage was not collected.

## Discussion

The results of a number of studies have led to the conclusion that periconceptional folic acid supplementation reduces the risk of NTDs [5-8]. Among the responses to this research evidence were calls in the early 1990s for mandatory fortification of food with folic acid. It was argued that this public health intervention would address concerns about achieving population level compliance with recommendations to women to take vitamin supplements containing folic acid before becoming pregnant and in the first weeks of pregnancy. These concerns were borne out in several Canadian studies suggesting that many caregivers [18,19] and women [20,21] remained unaware of the relationship between folic acid and NTDs. More recent studies have shown an increase in knowledge about folic acid, but supplementation rates remain low [22-25].

In March 1996 the US Food and Drug Administration (FDA) announced that it would permit addition of folic acid to enriched flour and other enriched cereal grain products, and that this addition would be mandatory as of January 1998. The level of fortification was set at 0.14 mg folic acid per 100 g of cereal grain product. It was determined that at this level of fortification, the intake of folate (from all sources) for the target and the general population would be kept below 1,000 μg/day, which was deemed to be the safe upper limit. This level of fortification was estimated to increase the average daily intake of folic acid in women of childbearing age by about 100 μg [26]. Subsequent to the US decision, Canada followed suit, permitting folic acid fortification at an equivalent level in December 1996 (addition of folic acid to white flour and enriched pasta and cornmeal at 0.15 mg folic acid per 100 g of flour and 0.20 mg folic acid per 100 g of pasta). In Canada, fortification became mandatory in November 1998.

### Rate of NTDs

Our results show a highly significant drop in the rate of NTDs in Newfoundland, taking into account all identified affected pregnancies (live births, stillbirths and pregnancies terminated after a prenatal diagnosis of an NTD). The 78% (95% CI 65%–86%) reduction in the NTD rate after implementation of fortification is greater than the 18%–22% reduction predicted at current levels of fortification [27,28], and greater than the 19% reduction in birth prevalence of NTDs reported in the US after mandatory fortification [29]. The results in Newfoundland are closer to the 54% reduction (95% CI 34%–68%) in rate of NTDs reported in Nova Scotia after fortification [2]. De Wals et al. observed a 32% reduction (95% CI 23%–41%) in NTDs in Quebec between 1992–97 and 1998–2000 [30]. Ray et al. [31] analyzed maternal serum screening data for Ontario and observed a decline in NTD prevalence from 1.13 per 1,000 pregnancies before fortification to 0.58 per 1,000 pregnancies thereafter (prevalence ratio 0.52, 95% CI 0.40–0.67).

The large reduction in the rate of NTDs in Newfoundland may be due, at least in part, to the fact that Newfoundland had higher background rates of NTDs. This population may be more sensitive to the influence of folic acid. In a large-scale public health campaign in northern and southern China, periconceptional use of 400 μg/day folic acid supplements was associated with a reduction in NTD risk of 79% for women in northern China, where the baseline NTD rate was high and similar to that observed in Newfoundland. A lower risk reduction of 41% was observed in the southern region where the pre-campaign NTD rate was much lower [32].

The 65% increase in the proportion of women taking vitamin supplements containing folic acid, from 17% in Phase I to 28% in Phase II, suggests that an increasing trend in folic acid supplementation may have played a role in the declining NTD rate in Newfoundland. In this study it was not possible to determine the individual contribution of supplementation and fortification to the trend in NTDs.

The annual rate of NTDs in the pre-fortification period (1994–97) did not differ significantly from that of the pre-supplementation period (1991–93); this is true whether 1997 is excluded or included in the pre-fortification period. The increase in the rates of NTDs in 1995 and 1996 appears random and largely unexplainable. The changes in the NTD rates between 1994–1996 and 1991–1993 and between 1995–1997 and 1991–1993 were not statistically significant. In addition, our data do not show an obvious increase in the proportion of NTDs in terminated pregnancies during 1994 and 1996 (data available upon request).

### Dietary intakes and blood folate levels

The questionnaire used in this dietary assessment was a modified Willett questionnaire [16], administered in a face-to-face interview with trained personnel. The Willett food frequency dietary questionnaire has been well validated [33] and proved easy to administer for this sample population.

The daily intake of naturally occurring folate among women aged 19–44 years in this study (average 248 μg/day in the Phase II sample) was similar to values found in other studies of women's diet [34,35]. For seniors in Phase II, naturally occurring folate in the diet averaged 290 μg/day which was comparable to values found for persons age 49 and older in an Australian study [36]. The dietary folic acid intake due to fortification did not exceed the Tolerable Upper Intake Level (UL) of 1,000 μg folic acid/day [14] for any of the participants (this UL for folic acid does not include naturally occurring folate). It is important to note that this part of the study excluded persons taking vitamin supplements containing folic acid. While it was not possible to estimate the proportion of people in the general Newfoundland population who may be consuming more than 1,000 μg/day of folic acid from fortification and supplementation combined, it is likely that this proportion is small. The average dietary intake and maximum intake of folic acid due to fortification were 70 μg/day and 235 μg/day, respectively, for women aged 19–44 years, and 74 μg/day and 219 μg/day, respectively, for seniors. The average folic acid dose in folic acid containing over-the-counter supplements marketed in Canada is about 350 μg/day (Health Canada unpublished information).

The results of this study provide strong evidence of improved blood folate status in women aged 19–44 years following mandatory fortification with folic acid. Women showed evidence of increased levels of serum and RBC folate and decreased levels of plasma HCY. These results are consistent with an earlier study examining the effect of fortification in the Framingham offspring study cohort [37].

Mandatory food fortification with folic acid has resulted in improvements in folate indices in seniors. Both mean serum folate and mean RBC folate increased following folic acid fortification (55% and 23%, respectively, Table 4). Consistent with this was a moderate decrease in mean plasma HCY levels among seniors by 1.3 μmol/L. Fortification of food with folic acid and an upward shift in blood folate levels is of benefit to the elderly population especially with regard to risk of cardiovascular disease. High levels of homocysteine are associated with both cerebrovascular and coronary heart disease [38-40].

### Vitamin B_12 _status

There was a decline in the proportion of seniors with low vitamin B_12 _levels, and there was actually a slight increase in mean vitamin B_12 _levels. In vitamin B_12 _deficiency, plasma MMA is usually elevated. Plasma MMA is believed to be a better indicator of vitamin B_12 _status at the tissue level than serum vitamin B_12 _levels are. Our study showed no change in mean MMA levels nor increased proportion of elderly with high levels. In addition, there was no change in the indicators of anaemia (i.e., haemoglobin and MCV) in seniors post fortification in our study. Thus, these results show no evidence of a deterioration in vitamin B_12 _status among seniors. Furthermore, there is no evidence of improved folate status resulting in masking of the haematological manifestations of vitamin B_12 _deficiency among seniors as a group. There was no evidence of deteriorating vitamin B_12 _status among young women participants based on vitamin B_12_ measurements. The upward trend in plasma MMA levels and higher proportion of abnormal values among young women is being further evaluated. It is unlikely that this is a direct effect of folic acid fortification and this observation is not consistent with any known effects of folic acid on vitamin B_12 _status.

### Limitations

We have documented the rate of NTDs among live births, stillbirths and terminated pregnancies known to have an NTD. It was not possible to include NTDs that may have occurred in pregnancies that resulted in a spontaneous abortion or a termination that occurred for reasons other than a congenital anomaly.

This study, and other studies of fortification in Canada, are limited by the fact that there was no precise date when exposure to food fortified with folic acid began. The addition of folic acid to white flour and enriched pasta and cornmeal was permitted as of December 1996. Industry was switching to folic acid-containing enrichment premixes, especially towards the end of 1997, in anticipation of both US requirements for fortification as of January 1, 1998, and Canadian plans to implement mandatory fortification. Although this requirement did not come into force in Canada until late 1998, the Phase I (November 1997 to March 1998) subjects of our study may have consumed at least some food fortified with folic acid. This would result in an underestimate of improvements in blood folate status due to fortification, and might lead us to miss adverse effects on vitamin B_12 _status. On the other hand, the fact that we observed such marked improvements in blood folate status leads us to conclude that there was a real increase in exposure to folic acid through fortification, over the study period.

Another limitation of this study is the possible underestimation of folic acid intake due to fortification. Our calculations were based on the assumption that manufacturers are fortifying flour at the required level. It has been suggested that allowance for "overages" is resulting in higher amounts in the affected products [41]. Also, for enriched pasta, the required level of fortification is from a minimum of 0.20 mg/100 g pasta to a maximum of 0.27 mg/100 g. In our calculations we assumed the minimum level of fortification.

We initially selected a random sample of subjects through random digit dialling, and asked eligible respondents for voluntary participation in the study. The reasonable level of response for the dietary questionnaire and blood sampling among rural and urban women aged 19–44 years suggests that with caution, we can generalize the results to all Newfoundland women of childbearing age. However, these findings may not be representative of the rest of Canada because of population differences in factors such as genetic background and dietary behaviour. These differences may also affect the generalizability of the NTD trend.

The sample response rate for the dietary questionnaire and blood sampling in seniors was approximately 45% both in Phase I and in Phase II. Many of the refusals to participate were due to illness of the eligible person. Furthermore, seniors residing in long term care settings were not included. Thus our sample population of seniors may be healthier than the general population age 65 and over in the province.

## Conclusions

The implementation of food fortification with folic acid has been accompanied by a marked decrease (78%) in the rate of NTDs in Newfoundland. The blood folate status of women aged 19–44 years improved following mandatory fortification. There is no evidence of adverse effects of the current levels of fortification on individuals aged 65 years and older. Specifically, there is no evidence to suggest an adverse effect of folic acid fortification on detection of abnormalities in vitamin B_12 _status based on biochemical and haematological indices.

Based on these findings, mandatory food fortification with folic acid should continue in Canada at the current levels. Over the time period of this study, the proportion of women aged 19–44 years taking a vitamin supplement containing folic acid increased. It was not possible to determine the magnitude of the separate contributions of fortification and supplementation to the decline in NTDs. Therefore, we recommend that public health efforts to promote awareness of the importance of folic acid supplementation among women of childbearing age continue. Ongoing surveillance of NTDs in Newfoundland and other parts of Canada is necessary to determine if the decline in NTD rate is maintained, and to enable further evaluation of prevention strategies. National surveillance of congenital anomalies including NTDs is a critical public health function that should be strengthened where necessary.

We look forward to the results of a current epidemiologic study, funded by the Canadian Institutes of Health Research (CIHR), of NTDs in 7 Canadian provinces between 1993 and 2002. There is also research into the relationship between increased folic acid consumption and reduced risk of other congenital anomalies, cardiovascular disease and cancer [42-45]. As this body of knowledge grows, public health practitioners and regulators in Canada and internationally will have more evidence with which to refine existing disease prevention policies and develop new ones.

## Competing interests

The authors declare that they have no competing interests.

## List of abbreviations

CBC, complete blood count

CI, confidence interval

CIHR, Canadian Institutes of Health Research

FDA, Food and Drug Administration

HCY, homocysteine

MCV, mean corpuscular volume

MMA, methylmalonic acid

NTD, neural tube defect

RBC, red blood cell

SPSS, Statistical Package for Social Sciences

## Authors' contributions

SL and CM oversaw the whole study and drafted the manuscript. SL carried out the analysis of NTD rate and statistical analysis. RW, LL, KSO and HS designed the study and carried out the data collection and the dietary assessment. ER, AL and VP participated in the design and carried out the blood analysis. MC carried out the collection of NTD data. All authors read, revised and approved the final manuscript.

## Pre-publication history

The pre-publication history for this paper can be accessed here:


